# Overview of data-synthesis in systematic reviews of studies on outcome prediction models

**DOI:** 10.1186/1471-2288-13-42

**Published:** 2013-03-16

**Authors:** Tobias van den Berg, Martijn W Heymans, Stephanie S Leone, David Vergouw, Jill A Hayden, Arianne P Verhagen, Henrica CW de Vet

**Affiliations:** 1 Department of Epidemiology and Biostatistics and the EMGO Institute for Health and Care Research, VU University Medical Centre, Amsterdam, The Netherlands; 2 Department of General Practice and the EMGO Institute for Health and Care Research, VU University Medical Centre, Amsterdam, The Netherlands; 3 Department of Community Health and Epidemiology, Dalhousie University, Halifax, Nova Scotia, Canada; 4 Department of General Practice, Erasmus Medical Centre, Rotterdam, The Netherlands

**Keywords:** Review, Systematic, Meta-analysis, Prediction, Prognosis, Forecasting, Methods

## Abstract

**Background:**

Many prognostic models have been developed. Different types of models, i.e. prognostic factor and outcome prediction studies, serve different purposes, which should be reflected in how the results are summarized in reviews. Therefore we set out to investigate how authors of reviews synthesize and report the results of primary outcome prediction studies.

**Methods:**

Outcome prediction reviews published in MEDLINE between October 2005 and March 2011 were eligible and 127 Systematic reviews with the aim to summarize outcome prediction studies written in English were identified for inclusion.

Characteristics of the reviews and the primary studies that were included were independently assessed by 2 review authors, using standardized forms.

**Results:**

After consensus meetings a total of 50 systematic reviews that met the inclusion criteria were included. The type of primary studies included (prognostic factor or outcome prediction) was unclear in two-thirds of the reviews. A minority of the reviews reported univariable or multivariable point estimates and measures of dispersion from the primary studies. Moreover, the variables considered for outcome prediction model development were often not reported, or were unclear. In most reviews there was no information about model performance. Quantitative analysis was performed in 10 reviews, and 49 reviews assessed the primary studies qualitatively. In both analyses types a range of different methods was used to present the results of the outcome prediction studies.

**Conclusions:**

Different methods are applied to synthesize primary study results but quantitative analysis is rarely performed. The description of its objectives and of the primary studies is suboptimal and performance parameters of the outcome prediction models are rarely mentioned. The poor reporting and the wide variety of data synthesis strategies are prone to influence the conclusions of outcome prediction reviews. Therefore, there is much room for improvement in reviews of outcome prediction studies.

## Background

The methodology for prognosis research is still under development. Although there is abundant literature to help researchers perform this type of research [1-5], there is still no widely agreed approach to building a multivariable prediction model [6]. An important distinction in prognosis is made between prognostic factor models, also called explanatory models and outcome prediction models [7,8]. Prognostic factor studies investigate causal relationships, or pathways between a single (prognostic) factor and an outcome, and focus on the effect size (e.g. relative risk) of this prognostic factor which ideally is adjusted for potential confounders. Outcome prediction studies, on the other hand, combine multiple factors (e.g. clinical and non-clinical patient characteristics) in order to predict future events in individuals, and therefore focus on absolute risks, i.e. predicted probabilities in logistic regression analysis. Methods that can be used to summarize data from prognostic factor studies in a meta-analysis can easily be found in the literature [9,10], but this is not the case for outcome prediction studies. Therefore, in the present study we focus on how authors of published reviews have synthesized outcome prediction models. The nomenclature to indicate various types of prognosis research is not standardized. We use prognosis research as an umbrella term for all research that might explain or predict a future outcome and prognostic factor and outcome prediction as specific types of prognosis studies.

In 2006, Hayden et al. showed that in systematic reviews of prognosis studies, different methods are used to assess the quality of primary studies [11]. Moreover, when quality is assessed, integration of these quality scores in the synthesis of the review is not guaranteed. For reviews of outcome prediction models, additional characteristics are important in the synthesis of models to reflect choices made in the primary studies, such as which variables are included in statistical models and how this selection was made. These choices therefore also reflect the internal and external validity of a model and influence the predictive performance of the model. In systematic reviews the researchers synthesize results across primary outcome prediction studies which include different variables and show methodological diversity. Moreover, relevant information is not always available, due to poor reporting in the studies. For example, several researchers have found that current knowledge about the recommended number of events per variable, and the coding and selection of variables, among other features, are not always reported in primary outcome prediction research [12-14]. Although improvement in primary studies themselves is needed, reviews that summarize outcome prediction evidence need to consider the current diversity in methodology in primary studies.

In this meta-review we focus on reviews of outcome prediction studies, and how they summarize the characteristics of design and analysis, and the results of primary studies. As there is no guideline nor agreement how primary outcome prediction models in medical research and epidemiology should be summarized in systematic reviews, an overview of current methods helps researchers to improve and develop these methods. Moreover, current methods for outcome prediction reviews are unknown to the research community. Therefore, the aim of this review was to provide an overview on how published reviews of outcome prediction studies describe and summarize the characteristics of the analyses in primary studies, and how the data is synthesized.

## Methods

### Literature search and selection of studies

Systematic reviews and meta-analyses of outcome prediction models that were published between October 2005 and March 2011 were searched. We were only interested in reviews that included multivariable outcome prediction studies. In collaboration with a medical information specialist, we developed a search strategy in MEDLINE, extending on the strategy used by Hayden [11], by adding recommended other search terms for predictive and prognostic research [15,16]. The full search strategy is presented in Appendix 1.

Based on title and abstract, potential eligible reviews were selected by one author (TvdB), who in case of any doubt included the review. Another author (MH) checked the set of potential eligible reviews. Ineligible reviews were excluded after consensus between both authors. The full texts of the included reviews were read, and if there was any doubt on eligibility a third review author (HdV) was consulted. The inclusion criteria were met if the study design was a systematic review with or without a meta-analysis, multiple variables were studied in an outcome prediction model, and the review was written in the English language. Reviews were excluded if they were based on individual patient data only, or when the topic was genetic profiling.

### Data-extraction

A data-extraction form was developed, based on important items to prognosis [1,2,12,13,17], to assess the characteristics of reviews and primary studies and is available from the first author on request. The items on this data-extraction form are shown in Appendix 2. Before the form was finalized it was pilot-tested by all review authors and minor adjustments were made after discussion about the differences in scores. One review author scored all reviews (TvdB) while other review authors (MH, AV, DV, and SL) collectively scored all reviews. Consensus meetings were held within 2 weeks after a review had been scored to solve disagreements. If consensus was not reached, a third reviewer (MH or HdV) was consulted to make a final decision.

An item was scored ‘yes’ if positive information was found about that specific methodological item, e.g. if it was clear that sensitivity analyses were conducted. If it was clear that a specific methodological requirement was not fulfilled, a ‘no’ was scored, e.g. no sensitivity analyses were conducted. In case of doubt or uncertainty, ‘unclear’ was scored. Sometimes, a methodological item could be scored as ‘not applicable’. The number of reviews within a specific answer category was reported, as well as the proportion.

## Results

### Literature search and selection process

**Figure 1:**
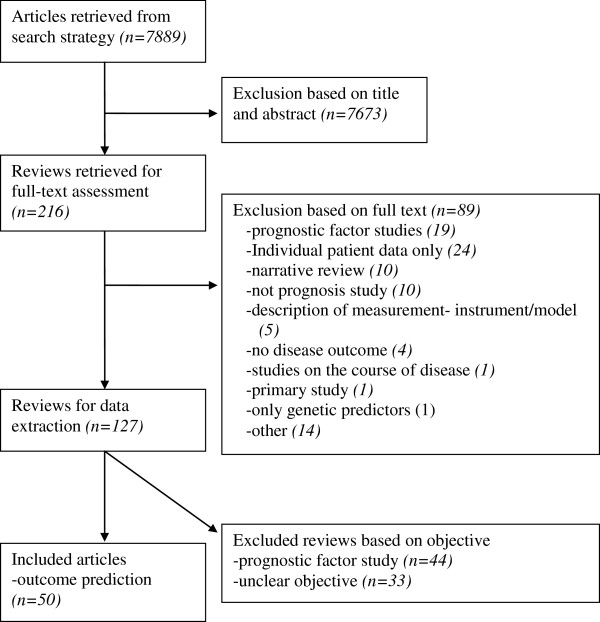
Flowchart of the search and selection process.

The search strategy revealed 7889 references and, based on title and abstract, 216 were selected to be read in full text (see the flowchart in Figure 1). Of these reviews, 89 were excluded and 127 remained. Exclusions after the full text had been read were mainly due to the focus of the research on a single variable with an outcome (prognostic factor study), analysis based on individual patient data only, or a narrative overview study design. After completing the data-extraction, the objectives and methods of 44 reviews described summaries of prognostic factor studies, and 33 reviews had an unclear approach. Therefore, a total of 50 reviews on outcome prediction studies were analyzed [18-67].

### Data-extraction

After completing the data-extraction form for all of the included reviews, most disagreements between review authors were found on items concerning the review objectives, the type of primary studies included, and the method of qualitative data-synthesis. Unclear reporting and, to a lesser degree, reading errors contributed to the disagreements. After consensus meetings only a small proportion of items needed to be discussed with a third reviewer.

### Objective and design of the review

Table 1, section 1 shows the items with regard to information about the reviews. Of the 50 reviews rated as summaries of outcome prediction studies, less than one third included only outcome prediction studies *[23,27,28,32,35,39,44,48],[50,52,55,58,60,66]. In about two thirds, the type of primary studies that were included was unclear, and the remaining reviews included a combination of prognostic factor and outcome prediction studies. Most reviews clearly described their outcome of interest. Also information about the assessment of the methodological quality of the primary studies, i.e. risk of bias, was provided in most reviews. In those that did, two thirds described the basic design of the primary studies in addition to a list of methodological criteria (defined in our study as a list consisting of at least four quality items). In some reviews an established criteria list was used or adapted, or a new criteria list was developed. In the reviews that assessed methodological quality, less than half actually used this information to account for differences in study quality, mainly by performing a ‘levels of evidence’ analysis, subgroup-analyses, or sensitivity analyses.

**Table 1:** Characteristics of the reviews and provided information about the included primary studies

				***n = 50 reviews***							
**Item:**	**Description of item:**			**Yes**		**No**		**Unclear**		**Not applicable**	
				**N**	**%**	**N**	**%**	**N**	**%**	**N**	**%**
**Section 1: Information about the objective and design of the reviews**											
1.	Type of primary studies included	**n = 50 (%)**									
	Only outcome prediction models	14	(28.0)								
	Combination of prognostic factor & outcome prediction studies	3	(6.0)								
	Unclear	33	(66.0)								
2.	Is the outcome of interest clearly described?			47	(94.0)	1	(2.0)	2	(4.0)		
3.	Is information about quality assessment provided?			36	(72.0)	14	(28.0)				
3a.	Method used										
	Methodological criteria list	3	(6.0)								
	Individual items	2	(4.0)								
	Not applicable	14	(28.0)								
	Methodological criteria & study design	31	(62.0)								
4.	Was study quality accounted for			21	(42.0)	13	(26.0)	2	(4.0)	14	(28.0)
4a.	Method used *#	**n = 23 (%)**									
	Exclusion of poor quality studies (cut-off score used)	3	(13.0)								
	Sensitivity analysis based on total quality score	5	(21.7)								
	Levels of evidence	12	52.2)								
	Subgroup analysis	7	(30.4)								
	Study findings weighted for quality	3	(13.0)								
	Other	2	(8.7)								
**Section 2: Information about the design and results of the primary studies**											
5.	Outcomes clearly described			36	(72.0)	20	(20.0)	4	(8.0)		
6.	Statistical methods used for variable selection described			2	(4.0)	46	(92.0)	2	(4.0)		
7.	Treatments described			6	(12.0)	37	(74.0)	7	(14.0)		
8.	Univariable point estimates for all the variables of the primary studies are provided			5	(10.0)	42	(84.0)	3	(6.0)		
8a.	Univariable estimates for dispersion for all the variables of the primary studies are provided			5	(10.0)	42	(84.0)	3	(6.0)		
9.	All variables (starting predictors) used to develop a model are described			4	(8.0)	36	(72.0)	10	(20.0)		
10.	Multivariable point estimates for each predictor in the final outcome prediction model are provided			11	(22.0)	33	(66.0)	4	(8.0)	2	(4.0)
10a.	Multivariable estimate of dispersion provided for each predictor in the final outcome prediction model			11	(22.0)	33	(66.0)	4	(8.0)	2	(4.0)
11.	Model performance is assessed and described			7	(14.0)	38	(76.0)	2	(4.0)	3	(6.0)
12.	number of events per variable is described			4	(8.0)	44	(88.0)	2	(4.0)		
**Section 3: Data-analysis and synthesis in the reviews**											
13.	Heterogeneity between studies described			45	(90.0)	4	(8.0)	1	(2.0)		
14.	Qualitative data-synthesis presented			49	(98.0)	1	(2.0)				
14a.	Method used	**n = 49 (%)**									
	Statistical significance	22	(44.9)								
	Consistency of findings	7	(14.3)								
	Consistency of findings & statistical significance	6	(12.2)								
	Available method of defining levels of evidence	3	(6.1)								
	Consistency of findings & levels of evidence	3	(6.1)								
	other combinations	8	(16.3)								
15.	Quantitative analysis performed			10	(20.0)	40	(80.0)				
15a.	Method used	**n = 10 (%)**									
	Random effects model	4	(40.0)								
	Fixed effects model	1	(10.0)								
	Random & Fixed effects model	3	(30.0)								
	Other	2	(20.0)								
				***n = 10 reviews***							
15b.	Statistical heterogeneity assessed			4	(40.0)	6	(60.0)				
15c.	Method used to assess statistical heterogeneity	**n = 4 (%)**									
	I^2^	2	(50.0)								
	I^2^ & Chi^2^	1	(25.0)								
	Other	1	(25.0)								
				***n = 50 reviews***							
16.	Graphic presentation of results provided			8	(16.0)	42	(84.0)				
16a.	Method used	**n = 8 (%)**									
	Forest plot	6	(75.0)								
	Forest plot & scatter plot	1	(12.5)								
	Barplot	1	(12.5)								
17.	Sensitivity analysis performed			6	(12.0)	43	(86.0)	1	(2.0)		
17a.	Method used	**n = 6 (%)**									
	Different cut-offs for study quality	3	(50.0)								
	Methodological criteria	1	(16.7)								
	Methodological criteria & weights for quality	1	(16.7)								
	Including other (excluded) cohorts	1	(16.7)								

* includes ‘yes’ and ‘unclear’ categories.

# numbers and percentages may add up to more than 23 and 100%, due to multiple methods in some reviews.

### Information about the design and results of the primary studies

In Table 1, section 2 shows information provided about the included primary studies. The outcome measures used in the included studies were reported in most of the reviews. Only 2 reviews [28,52] described the statistical methods that were used in the primary studies to select variables for inclusion of a final prediction model, e.g. forward or backward selection procedures, and 6 others whether and how patients were treated.

A minority of reviews [23,24,27,28] described for all studies the variables that were considered for inclusion in the outcome prediction model and only 5 reviews [36,37,39,48,55] reported univariable point estimates (i.e.. regression coefficients or odds ratios) and estimates of dispersion (e.g. standard errors) of all studies. Similarly, multivariable point estimates and estimates of dispersion were reported in respectively 11 and 10 of the reviews [21,26,27,31,33,37,44,52],[55,64,65].

With regard to the presentation of univariable and multivariable point estimates, 2 reviews presented both types of results [37,55], 31 did not report any estimates, and 17 reviews were unclear or reported only univariable or multivariable results [not shown in the table]. Lastly, model performance and number of events per variable were reported in 7 reviews [32,39,41,60,61,65,66] and 4 reviews [40,48,58,61], respectively.

### Data-analysis and synthesis in the reviews

Table 1, section 3 illustrates how the results of primary studies were summarized in the reviews. It shows that heterogeneity was described in almost all reviews by reporting differences in the study design and the characteristics of the study population. All but one review [57] summarized the results of included studies in a qualitative manner. Methods that were mainly used for that purpose were number of statistical significant results, consistency of findings, or a combination of these. Quantitative analysis, i.e. statistical pooling, was performed in 10 of the 50 reviews [25,28,31,36,37,44,45,57-59]. The quantitative methods used included random effects models and fixed effects models of regression coefficients, odds ratios or hazard ratios. Of these quantitative summaries, 40% assessed the presence of statistical heterogeneity using I^2^, Chi^2^, or the Q statistic. In two reviews [25,59], statistical heterogeneity was found to be present, and subgroup analysis was performed to determine the source of this heterogeneity [results not shown]. In 8 of the reviews there was a graphical presentation of the results, in which a forest plot [25,28,36-38,52,59], per single predictor, was the frequently used method. Other studies used a barplot [57] or a scatterplot [38]. In 6 reviews [25,26,32,43,46,58] a sensitivity analysis was performed to test the robustness of the choices made such as changing the cut-off value for a high or low quality primary study.

## Discussion

We made an overview of how systematic reviews summarize and report the results of primary outcome prediction studies. Specifically, we extracted information on how the data-synthesis was performed in reviews since outcome prediction models may consider different potential predictors, and include a dissimilar set of variables in the final prediction model, and use a variety of statistical methods to obtain an outcome prediction model.

Currently, in prognosis studies a distinction is made between outcome prediction models and prognostic factor models. The methodology of data synthesis in a review of the latter type of prognosis is comparable to the methodology of aetiological reviews. For that reason, in the present study we only focused on reviews of outcome prediction studies. Nonetheless, we found it difficult to distinct between both review types. Less than half of the reviews that we initially selected for data-extraction in fact seemed to serve an outcome prediction purpose. It appeared that the other reviews summarized prognostic factor studies only, or the objective was unclear. In particular, prognostic factor reviews that investigated more than one variable in addition to non-specific objectives made it difficult to clarify what the purpose of reviews was. As a consequence, we might have misclassified some of the 44 excluded reviews rated as prognostic factor. The objective of a review should also include information about the type of study that is included, that is of outcome prediction studies in this case. However, we found that in reviews aimed at outcome prediction the type of primary study was unclear for two-thirds of the reviews. An example we encountered in a review was that their purpose was “to identify preoperative predictive factors for acute post-operative pain and analgesic consumption” although the review authors included any study that identified one or more potential risk factors or predictive factors. The risk of combining both types of studies, i.e. risk factor or prognostic factor studies and predictive factor studies, is that inclusion of potential covariables in the former type are based on change in regression coefficient of the risk factor while in the latter study type all potential predictor variables are included based on their predictive ability of the outcome. This distinction may lead to: 1) biased results in a meta-analysis or other form of evidence synthesis because a risk factor is not always predictive for an outcome and 2) risk factor studies – if adjusted for potential confounders at all – have a slightly different method to obtain a multivariable model compared to outcome prediction studies which may also lead to biased regression coefficients. The distinction between prognostic factor and outcome prediction studies was already emphasized in 1983 by Copas [68]. He stated that “a method for achieving a good predictor may be quite inappropriate for other questions in regression analysis such as the interpretation of individual regression coefficients”. In other words, the methodology of outcome prediction modelling differs from that of prognostic factor modelling, and therefore combining both types of research into one review to reflect current evidence should be discouraged. Hemingway et al. [2] appealed for standard nomenclature in prognosis research, and the results of our study underline their plea. Authors of reviews and primary studies should clarify their type of research, for example by using the terms applied by Hayden et al. [8] ‘prognostic factor modelling’ and ‘outcome prediction modelling’, and give a clear description of their objective.

Studies included in outcome prediction reviews are rarely similar in design and methodology, and this is often neglected when summarizing the evidence. Differences, for instance in the variables studied and the method of analysis for variable selection might explain heterogeneity in results, and should therefore be reported and reflected on when striving to summarize evidence in the most appropriate way. There is no doubt that the methodological quality of primary studies included in reviews is related to the concept of bias [69,70] and it is therefore important to assess this [11,69,70]. Dissemination bias reflects if publication bias is likely to be present, how this is handled and what is done to correct for it [71]. To our knowledge, dissemination bias and especially its consequences in reviews of outcome prediction models are not studied yet. Most likely testimation bias [5], i.e. the predictors considered and the amount of predictors in relation to the effective sample size influence results more then publication bias. Therefore, we did not study dissemination bias on the review level.

With regard to the reporting of primary study characteristics in the systematic reviews, there is much room for improvement. We found that the methods of model development (e.g. the variables considered and the variable selection methods used) in the primary studies were not, or only vaguely reported in the included reviews. These methods are however important, because variable selection procedures can affect the composition of the multivariable model due to estimation bias, or may result in an increase in model uncertainty [72-74]. Furthermore, the predictive performance of the model can be biased by these methods [74]. We also found that only 5 of the reviews reported what kind of treatment the patients received in the primary studies. Although prescribed treatment is often not considered as a candidate predictor, it is likely to have a considerable impact on prognosis. Moreover, treatment may vary in relation to predictive variables [75], and although randomized controlled trials provide patients with similar treatment strategies, in cohort studies which are most often seen in prognosis research this is often not the case. Regardless of difficulties in defining groups that receive the same treatment, it is imperative to consider treatment in outcome prediction models. In order to ensure correct data-synthesis of the results, the primary studies not only should provide point estimates and estimates of dispersion of all the included variables, but also for non-significant findings. Whereas the results of positive or favourable findings are more often reported [75-78], the effects of predictive factors that do not reach statistical significance also need to be compared and summarized in a review. Imagine a variable being of statistical significance in one article, but not reported in others because of non-significance. It is likely that this one significant result is a spurious finding or that the others were underpowered. Without information about the non-significant findings in other studies, biased or even incorrect conclusions might be drawn. This means that reporting of the evidence of primary studies should be accompanied by the results of univariable and multivariable associations, regardless of their level of significance. Moreover, confidence intervals, or other estimates of dispersion are also needed in the review, and unfortunately these results were not presented in most of the reviews in our study. Some reviews considered differences in unadjusted and adjusted results, and the results of one review were sensibly stratified according to univariable and multivariable effects [38]. Other reviews merely reported multivariable results [31], or only univariable results if multivariable results were unavailable [58]. In addition to the multivariable results of a final prediction model, the predictive performance of these models is important for the assessment of clinical usefulness [79]. A prediction model in itself does not indicate how much variance in outcome is explained by the included variables. Unfortunately, in addition to the non-reporting of several primary study characteristics, the performance of the models was rarely reported in the reviews included in our overview.

Different stages can be distinguished in outcome prediction research [80]. Most outcome prediction models evaluated in the systematic reviews appeared to be in a developmental phase. Before implementation in daily practice, confirmation of the results in other studies is needed. With this type of validation studies underway, in future reviews we should acknowledge the difference between externally validated models and models from developmental studies, and analyze them separately.

In systematic reviews data can be combined quantitatively, i.e. a meta-analysis can be performed. This was done in 10 of the reviews. All of them combined point estimates (mostly odds ratios, but also a mix of odds ratios, hazard ratios and relative risks) and confidence intervals for single outcome prediction variables. This made it possible to calculate a pooled point estimate, often complemented with confidence intervals [81]. However, in outcome prediction research we are interested in the estimates of a combination of predictive factors, which makes it possible to calculate absolute risks or probabilities to predict an outcome in individuals [82]. Even if the relative risk of a variable is statistically significant, it does not provide information about the extent to which this variable is predictive for a particular outcome. The distribution of predictor values, outcome prevalence, and correlations between variables also influences the predictive value of variables within a model [83]. Effect sizes also provide no information about the amount of variation in outcomes that is explained. In summary: the current quantitative methods seem to be more of an explanatory way of summarizing the available evidence, instead of quantitatively summarizing complete outcome prediction models.

Medline was the only database that was searched for relevant reviews. Our intention was to provide an overview of recently published reviews and not to include all relevant outcome prediction reviews. Within Medline, some eligible reviews may have been missed if their titles and abstracts did not include relevant terms and information. An extensive search strategy was applied and abstracts were screened thoroughly and discussed in case of disagreement. Data-extraction was performed in pairs to prevent reading and interpretation errors. Disagreements mainly occurred when deciding on the objective of a review and the type of primary studies included, due to poor reporting in most of the reviews. This indicates a lack of clarity, explanation and reporting within reviews. Therefore, screening in pairs is a necessity, and standardized criteria should be developed and applied in future studies focusing on such reviews. Consistency in rating on the data-extraction form was enhanced by one review author rating all reviews, with one of the other review authors as second rater. Several items were scored as “no”, but we did not know whether this was a true negative (i.e. leading to bias) or that no information was reported about a particular item. For review authors it is especially difficult to summarize information about primary studies because there may be a lack of information in the studies [13,14,84].

### Implications

There is still no available methodological procedure for a meta-analysis of regression coefficients of multivariable outcome prediction models. Some authors, such as Riley et al. and Altman [81,84], are of the opinion that it remains practically impossible, due to poor reporting, publication bias, and heterogeneity across studies. However, a considerable number of outcome prediction studies have been published, and it would be useful to integrate this body of evidence into one summary result. Moreover, there is an increase in the number of reviews that are being published. Therefore, there is a need to find the best strategy to integrate the results of primary outcome prediction studies. Consequently, until a method to quantitatively synthesize results has been developed, a sensible qualitative data-synthesis, which takes methodological differences between primary studies into account, is indicated. In summarizing the evidence, differences in methodological items and model-building strategies should be described and taken into account when assessing the overall evidence for outcome prediction. For example, univariable and multivariable results should be described separately, or subgroup analyses should be performed when they are combined. Other items that, in our opinion should be taken into consideration with regard to the data-synthesis are: study quality, variables used for model development, statistical methods used for variable selection procedures, the performance of models, and sufficient cases and non-cases to guarantee adequate study power. Regardless of whether or not these items are taken into consideration in the data-synthesis, we strongly recommend that in reviews they are described for all primary studies included so that readers can also take them into consideration.

## Conclusion

In conclusion, poor reporting of relevant information and differences in methodology occur in primary outcome prediction research. Even the predictive ability of the models was rarely reported. This, together with our current inability to pool multivariable outcome prediction models, challenges review authors to make informative reviews of outcome prediction models.

## Appendix 1

Search strategy: 01-03-2011

Database: MEDLINE

((“systematic review”[tiab] OR “systematic reviews”[tiab] OR “Meta-Analysis as Topic”[Mesh] OR meta-analysis[tiab] OR “Meta-Analysis”[Publication Type]) AND (“2005/11/01”[EDat] : “3000”[EDat]) AND ((“Incidence”[Mesh] OR “Models, Statistical”[Mesh] OR “Mortality”[Mesh] OR “mortality ”[Subheading] OR “Follow-Up Studies”[Mesh] OR “Prognosis”[Mesh:noexp] OR “Disease-Free Survival”[Mesh] OR “Disease Progression”[Mesh:noexp] OR “Natural History”[Mesh] OR “Prospective Studies”[Mesh]) OR ((cohort*[tw] OR course*[tw] OR first episode*[tw] OR predict*[tw] OR predictor*[tw] OR prognos*[tw] OR follow-up stud*[tw] OR inciden*[tw]) NOT medline[sb]))) NOT ((“addresses”[Publication Type] OR “biography”[Publication Type] OR “case reports”[Publication Type] OR “comment”[Publication Type] OR “directory”[Publication Type] OR “editorial”[Publication Type] OR “festschrift”[Publication Type] OR “interview”[Publication Type] OR “lectures”[Publication Type] OR “legal cases”[Publication Type] OR “legislation”[Publication Type] OR “letter”[Publication Type] OR “news”[Publication Type] OR “newspaper article”[Publication Type] OR “patient education handout”[Publication Type] OR “popular works”[Publication Type] OR “congresses”[Publication Type] OR “consensus development conference”[Publication Type] OR “consensus development conference, nih”[Publication Type] OR “practice guideline”[Publication Type]) OR (“Animals”[Mesh] NOT (“Animals”[Mesh] AND “Humans”[Mesh]))).

## Appendix 2

Items used to assess the characteristics of analyses in outcome prediction primary studies and reviews:

Information about the review:

1. What type of studies are included?

2. Is(/are) the outcome(s) of interest clearly described?

3. Is information about the quality assessment method provided?

a. What method was used?

4. Did the review account for quality?

a. What method was used?

Information about the analysis of the primary studies:

5. Are the outcome measures clearly described?

6. Is the statistical method used for variable selection described?

7. Is there a description of treatments received provided?

Information about the results of the primary studies:

8. Are crude univariable associations and estimates of dispersion for all the variables of the primary studies presented?

9. Are all variables that were used for model development described?

10. Are the multivariable associations and estimates of dispersions presented?

11. Is model performance assessed and reported?

12. Is the number of predictors relative to the number of outcome events described?

Data-analysis and synthesis of the review:

13. Is the heterogeneity of primary studies described?

14. Is a qualitative synthesis presented?

a. What method was used?

15. Are methods for quantitative analysis described?

a. What method was used?

b. Is the statistical heterogeneity assessed?

c. What method is used to assess statistical heterogeneity?

d. If statistical heterogeneity exists, are sources of the heterogeneity investigated?

e. What method is used to investigate potential sources of heterogeneity?

16. Is a graphical presentation of the results provided?

a. What method was used?

17. Are sensitivity analysis performed?

a. On which level?

## Competing interests

All authors report no conflicts of interests.

## Authors’ contributions

TvdB, had full access to all of the data in the study and takes responsibility for the integrity of the data and the accuracy of the data analysis. *Study concept and design:* TvdB, MH, JH, AV, HdV. *Acquisition of data:* TvdB, MH, SL, DV, AV, HdV *Analysis and interpretation of data:* TvdB, MH, HdV. *Drafting of the manuscript:* TvdB, MH, HdV. *Critical revision of the manuscript for important intellectual content:* TvdB, MH, SL, DV, JH, AV, HdV. *Statistical analysis:* TvdB *Study supervision:* MH, HdV. All authors read and approved the final manuscript.

## Pre-publication history

The pre-publication history for this paper can be accessed here:

http://www.biomedcentral.com/1471-2288/13/42/prepub
